# Obstructive Giant Inflammatory Polyposis as a First Manifestation of Crohn’s Disease: A Case Report

**DOI:** 10.3390/healthcare10101995

**Published:** 2022-10-11

**Authors:** Andre Bratanic, Bruna Rosic Despalatovic, Berna Pavic, Tina Bozikovic, Zarko Ardalic, Danijel Antonio Grubisic, Katarina Vilovic

**Affiliations:** 1 Department of Gastroenterology, University Hospital Split, 21000 Split, Croatia; 2 “J&J MEDICI” Polyclinic for Internal Medicine, 21000 Split, Croatia; 3 School of Medicine, University of Split, 21000 Split, Croatia; 4 Department of Pathology, University Hospital Split, 21000 Split, Croatia

**Keywords:** giant inflammatory polyps, inflammatory bowel diseases, Crohn’s disease, colonic obstruction

## Abstract

Colonic inflammatory polyps (pseudopolyps) are common feature of inflammatory bowel diseases. They usually do not grow excessively, rarely reaching more than 15 mm in size, at which point they are termed giant inflammatory polyps. Clinical presentation of these polyps can vary greatly, ranging from being completely asymptomatic, usually detected incidentally, to abdominal cramps, rectal bleeding or intestinal obstruction. More importantly, giant inflammatory polyps can be easily mistaken for colonic malignancy, although without having malignant potential themselves. These polyps rarely regress with successful medical treatment of inflammatory bowel diseases and often require surgical treatment. We present an unusual case of giant inflammatory polyps which was the first presentation of inflammatory bowel disease. It was initially mistaken for colonic malignancy with intestinal obstruction, which led to surgical treatment.

## 1. Introduction

The development of post-inflammatory polyps, usually termed “pseudopolyps”, is relatively common feature in inflammatory bowel diseases [1]. In rare cases, these polyps form large agglomerations of filiform projections; if they measure >15 mm in any direction, they are defined as giant inflammatory polyps [2,3]. The probable pathogenetic basis of such an entity is repeated episodes of inflammation and healing within the colonic surface, with the formation of polypoid projections of residual mucosa amidst surrounding ulcerated areas. Giant inflammatory polyps occur in both ulcerative colitis and Crohn’s disease patients [2]. Given their clinical, endoscopic and radiologic appearance, giant inflammatory polyps can easily be mistaken for colorectal carcinoma [4]. Here we present a case of a giant inflammatory polyp as the first manifestation of inflammatory bowel disease. It was initially mistaken for colonic cancer, and its histopathologic features regarding inflammatory bowel disease phenotype proved to be ambiguous at the beginning.

## 2. Case Report

A 55-year-old patient was urgently hospitalized due to bowel obstruction symptoms. The patient had no previous history of gastrointestinal diseases, nor any other chronic condition. Based on the MSCT results, an obstructive malignancy of the colon was suspected, and an urgent right-side hemicolectomy with an ileotransversostomy was performed. Histopathologic analysis of the resected bowel segment showed polypoid changes in the colonic surface with signs of chronic inflammation, low-grade dysplasia, dilated branching crypts and increased lamina propria cellularity. An excess of histiocytes in the lamina propria, muscularis mucosa and serosa was also found. The final histopathologic diagnosis was a pseudopolyposis of the colon within ulcerative colitis.

**Figure 1:**
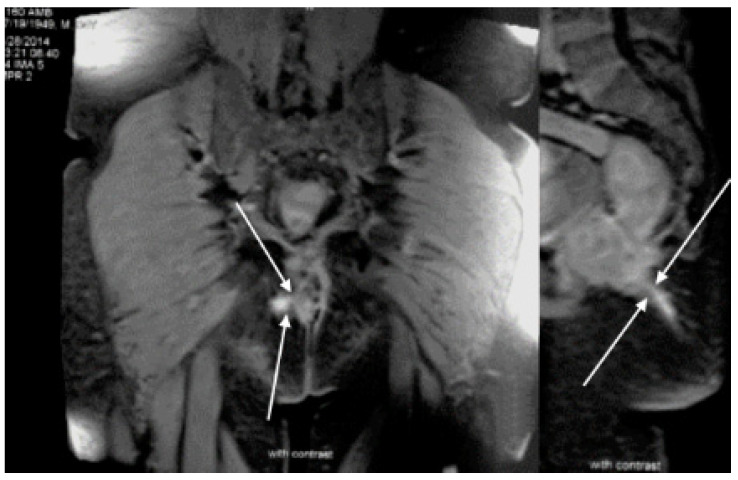
Pelvic MR: Arrows show complicated, branching perianal cutaneous fistula.

**Figure 2:**
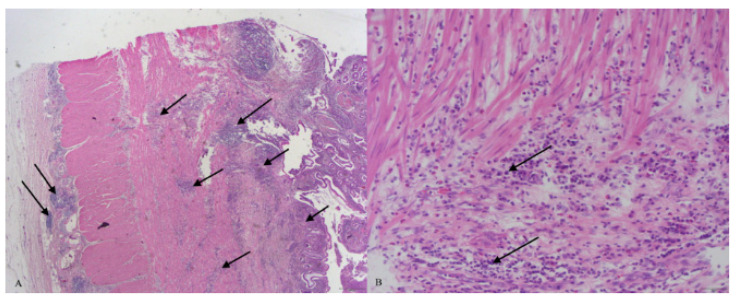
Histopathologic analysis of surgical specimen of colon. Arrows show leukocyte infiltration (**A**) HE 20×, transmural inflammation on surgical specimen; (**B**) HE 200×, inflammation cell infiltration of the muscular layer.

After hemicolectomy, a treatment with mesalamine was introduced, and the patient was not seen for the next seven years. Allegedly, he did not have any symptoms during that time, and he stopped taking mesalamine after a year. Seven years after surgery, an episode of diarrhea, weight loss, subfebrility and weakness appeared, lasting for a few months. A perianal fistula also appeared at the same time. An extensive diagnostic work-up was conducted, including laboratory, endoscopic, histopathologic and radiologic assessment. On a CT scan, a wall thickening of the entire colon and neoterminal ileum was seen. Endoscopically, both the colonic and ileic mucosa were edematous and covered with ulcers, while several giant inflammatory polyps were found in the colon, at the level of the splenic flexure. A colonic biopsy was repeated, and histopathologic analysis confirmed that active chronic inflammation was present. Because a perianal fistula had developed (Figure 1), a revision of the previous (postsurgical) histopathologic finding was performed. An inflammation affecting muscular and serous layers of colon was seen in the revised histopathologic analysis, and a new, definite diagnosis of Crohn’s disease was established (Figure 2). An induction therapy was initiated, including systemic corticosteroids and mesalamine, along with antibiotics, and soon a clinical remission was achieved. Azathioprine was added as maintenance therapy, and subsequently, corticosteroids were tapered down and then excluded.

After a year, a Crohn’s disease exacerbation occurred, and corticosteroids were reintroduced, along with the introduction of infliximab. On this therapy, a clinical remission was achieved in a couple of months, and it was further maintained with infliximab, after corticosteroid withdrawal.

**Figure 3:**
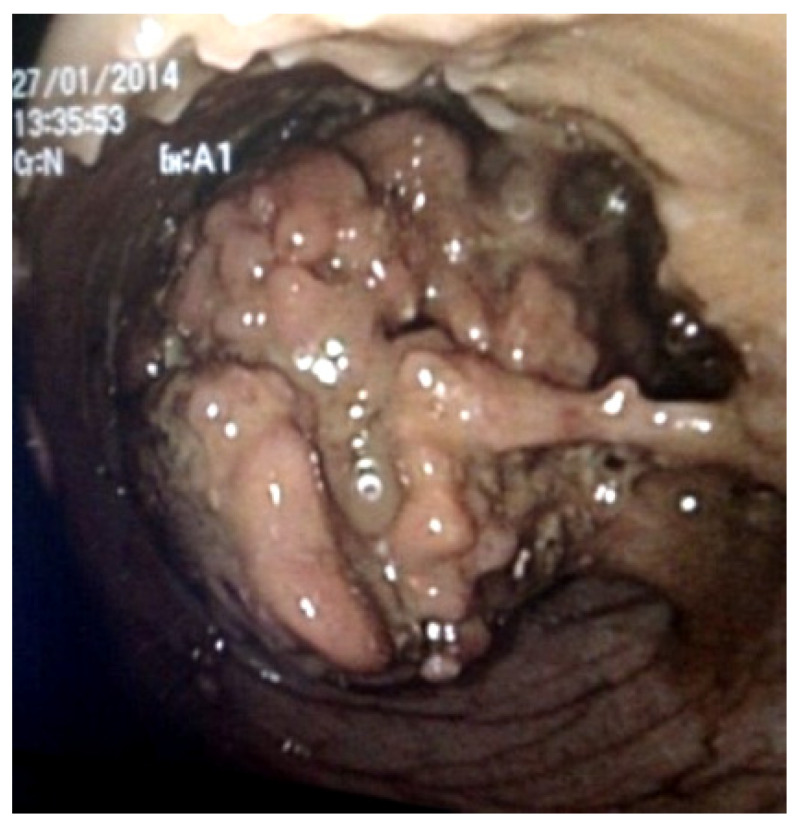
Endoscopic view of conglomerate of giant inflammatory polyps on the splenic flexure of the colon.

A year later, there was still an active perianal fistula. Endoscopy revealed normal colonic mucosa distal to the splenic flexure, while on the flexure, there was a conglomerate of giant inflammatory polyps’ (Figure 3).

**Figure 4:**
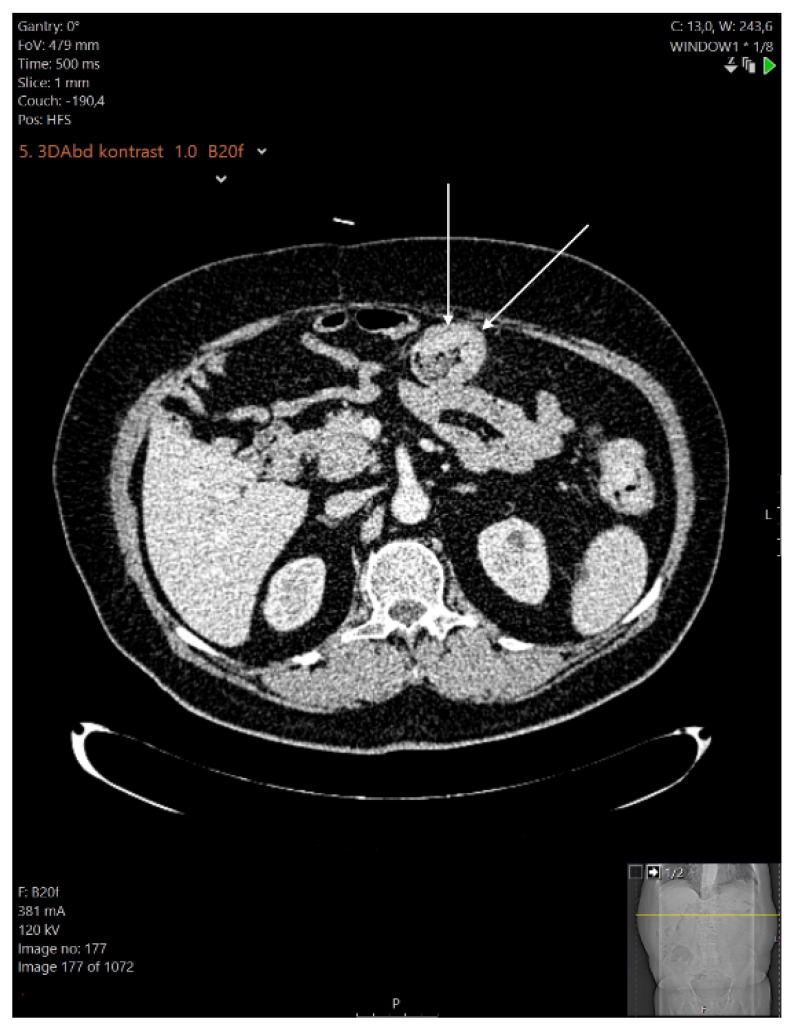
Abdominal multislice CT scan: colonic wall thickening with giant inflammatory polyps.

**Figure 5:**
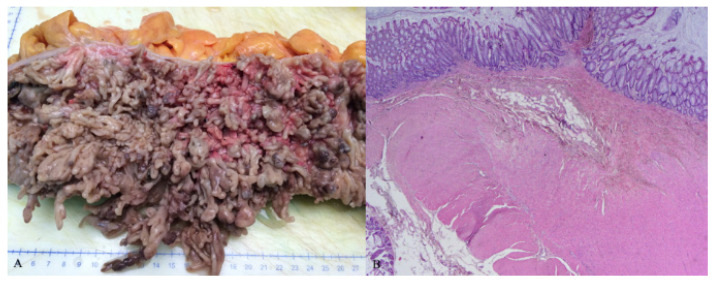
(**A**) Macroscopic view of surgical specimen of colon with giant inflammatory polyps; (**B**) HE 20×, microscopic view of colonic specimen without transmural inflammation.

It was decided to continue the therapy with infliximab, and the perianal fistula was successfully treated surgically with seton, along with antibiotics. Regardless of the endoscopic and biochemical remission of the underlying disease achieved with infliximab, after six months the patient began to experience bowel obstruction symptoms due to giant inflammatory polyps (Figure 4). Therefore, it was decided to treat the obstruction surgically, and a subtotal colectomy followed. A histopathologic analysis of the surgical specimen showed extensive pseudopolyposis, but inflammation was present only in the mucosal layer of the colon and did not affect the muscular layer (Figure 5).

Postsurgically, treatment with infliximab and mesalamine was continued. The patient was controlled on regular basis; there has been no further disease exacerbation, and no new giant inflammatory polyps have been found on endoscopy so far.

## 3. Discussion

### 3.1. Development of Pseudopolyps

One of the most important features of inflammatory bowel diseases is their remitting clinical course, with repeating episodes of exacerbations and remissions [2]. As a result, post-inflammatory polyps (pseudopolyps) develop in approximately 10–20% of inflammatory bowel disease patients [1].

Pseudopolyps can present as solitary or multiple, and either localized or diffuse in terms of distribution [5]. They can also be of various sizes, but they are usually small. If they exceed 15 mm in size, they are defined as giant inflammatory polyps [6]. The description of this entity first appeared in the literature in 1965 [5]. There is also a distinct form of pseudopolyps: thin, finger-like projections of mucosa and submucosa called filiform polyps. They usually form a cluster and are often also referred to as giant inflammatory polyps [7,8].

Regarding the prevalence of colonic giant pseudopolyposis, the results of an early study by Kelly et al. showed that among colectomized patients with giant inflammatory polyps, 66.6% had Crohn’s disease, and 33.7% had ulcerative colitis [9]. However, a more recent study by Maggs et al. found a prevalence of giant inflammatory polyps of 53.8% in ulcerative colitis patients and 46.2% in Crohn’s disease patients [3].

The development of giant inflammatory polyps can be partially explained by recently obtained knowledge on the pathophysiology of autoimmune diseases. It was found that Th17 cells are responsible for different inflammatory and autoimmune response mechanisms. IL-17 may intensify local inflammation by promoting angiogenesis and increase the production and expression of vascular endothelial growth factor (VEGF) in autoimmune diseases such as rheumatoid arthritis. Studies performed with bowel biopsies show that IL-23, IL-17 and IL-12 are overexpressed in mucosal lesions of Crohn’s disease, suggesting that these models of autoimmunity are linked to Th17 and not primarily to Th1 responses [10].

Because giant inflammatory polyps develop in postinflamed and scarred areas of the colon, histopathologic changes are often transmural, similar to those seen in Crohn’s disease. This could pose a pitfall for a pathologist; however, to confirm the diagnosis of Crohn’s disease, a transmural inflammation has to be found in areas of the colon beyond those affected by pseudopolyposis [11]. In the case we present, given that our patient had not a history of inflammatory bowel disease prior to presentation, and on the basis of clinical and histopathologic consideration, an initial diagnosis of ulcerative colitis was established. Only later in the clinical course, with disease exacerbation affecting the ileum and with development of perianal fistulas, was the histopathologic finding revised and a definitive diagnosis of Crohn’s disease established.

### 3.2. Clinical Presentation

There is no clear trend in terms of an increased prevalence of pseudopolyps with extended periods of history of inflammatory bowel disease in early studies. Jalan et al. reported that 33% of patients with pseudopolyps had a history of ulcerative colitis shorter than 5 months, while De Dombai et al. found that 8.8% of patients with ulcerative colitis had pseudopolyps on the first flare-up of the disease [12,13]. However, when giant pseudopolyposis is discussed, its appearance is usually associated with severity and longer duration of inflammatory bowel disease [3,14]. In the study of Ooi et al., the median disease history from diagnosis to the appearance of giant inflammatory polyps was 5 years for ulcerative colitis and 6 years for Crohn’s disease [15]. On the other hand, results from the study of Yada et al. showed a great range of time between the initial diagnosis of inflammatory bowel disease and the diagnosis of giant inflammatory polyps, ranging from 3 to 276 months, with the possibility of development of giant pseudopolyp early in the course of the disease, as in our case, where it was the first manifestation of the disease [16].

Regarding the association between giant inflammatory polyps and the severity of the disease, it was noted that it can develop in both active and inactive phases of the disease, as shown in the study of Abou Rached et al. [14]. In the case we present, the first giant inflammatory polyp appeared during the active phase of the disease, while a recidivant one grew in size and finally caused a total colonic obstruction despite clinical remission having been achieved. The clinical presentation of giant inflammatory polyps can also vary significantly, depending on size and localization. Several studies have revealed that giant inflammatory polyps most commonly develop in the transverse colon, followed by the descending and sigmoid colon, with the rectum being the least common site; thus, they follow the distribution pattern of colonic pseudopolyps in general [3,13,17]. In our case, the initial giant inflammatory polyps were found only in the right (ascendent) colon, according to MSCT, while the recidivant one was found at the splenic flexure.

While pseudopolyps rarely cause serious complications, the situation is different with giant inflammatory polyps, which warrant awareness on the part of physicians. Maggs et al. reported that among 78 patients with giant inflammatory polyps, 15% were complicated by obstruction or sub-obstruction, and 3% by intussusception. In addition, giant inflammatory polyps can produce symptoms similar to those of inflammatory bowel diseases, including bloating, diarrhea and abdominal pain. In the same study, from among the total of 25 patients with inactive disease, 11 had symptoms that regressed after removal of the giant inflammatory polyps [3].

In the case of excessive growth, giant inflammatory polyps can easily be mistaken for a malignant colonic obstruction [4,17]. Such a misguidance is especially possible when obstruction is the first clinical manifestation of the giant inflammatory polyps, as was the case for our patient, where it required an urgent right hemicolectomy. Moreover, in the further clinical course, our patient developed yet another intestinal obstruction, which resulted in subtotal colectomy. However, even in patients with a previously known history of inflammatory bowel disease, suspicion of malignant obstruction would be feasible with respect to an increased incidence of colonic carcinoma in inflammatory bowel disease patients [18]. On the other hand, there is only one case reported in the literature of carcinoma developing in giant inflammatory polyps [19].

### 3.3. Treatment

Data on the treatment of giant inflammatory polyps are generally scarce. Conservative (medical), endoscopic or surgical methods can be used. In patients with asymptomatic giant inflammatory polyps or in those with symptoms of inflammatory bowel disease only, adequate medical treatment of inflammatory bowel disease is warranted [1,3]. There are reports indicating the regression of giant inflammatory polyps upon successful treatment of inflammatory bowel disease with mesalamine, azathioprine and infliximab [2,20].

In symptomatic patients, however, interventional treatment (endoscopic or surgical) is required [21].

Coagulation with argon plasma or laser ablation can be applied in the case of bleeding giant inflammatory polyps [22,23]. Endoscopic resection or polypectomy can also be performed in the case of symptomatic giant inflammatory polyps [24,25]. Finally, surgical methods can be used if endoscopy fails, especially in patients with colonic obstruction [26].

Regarding our case, the patient was initially treated surgically due to obstruction symptoms and suspicion of malignancy. After the diagnosis of inflammatory bowel disease (ulcerative colitis) had been established, he was treated accordingly (mesalamine). Once a recurrent giant inflammatory polyp had been diagnosed, along with exacerbation of inflammatory bowel disease (now Crohn’s disease, according to the revised diagnosis), the treatment was intensified, including several therapeutic lines (corticosteroids, azathioprine and infliximab). Consequently, a remission of the underlying disease was achieved, but there was no regression of the giant inflammatory polyps. Finally, once a colonic obstruction had developed, a subtotal colectomy was performed.

In conclusion, we can say that giant inflammatory polyps represent a rare complication of inflammatory bowel diseases with specific clinical and histological features that can sometimes pose a diagnostic and therapeutic perplexity for a clinician. Therefore, close cooperation between gastroenterologist, pathologist and surgeon is essential for the proper management of this type of lesion.

## Data Availability

No new data were created or analyzed in this study. Data sharing is not applicable to this article.
